# De-Esterified Tragacanth Microspheres Loaded into Eudragit S-100 Coated Capsules for Colon-Targeted Delivery

**Published:** 2018

**Authors:** Ehsan Ahmadi, Komail Sadrjavadi, Ghobad Mohammadi, Ali Fattahi

**Affiliations:** a *Student Research Committee, Kermanshah University of Medical Sciences, Kermanshah, Iran.*; b *Pharmaceutical Sciences Research Center, School of Pharmacy, Kermanshah University of Medical Sciences, Kermanshah, Iran.*; c *Nano Drug Delivery Research Center, Faculty of Pharmacy, Kermanshah University of Medical Sciences, Kermanshah, Iran. *; d *Medical Biology Research Center, Kermanshah University of Medical Sciences, Kermanshah, Iran.*

**Keywords:** De-esterified tragacanth, Microsphere, Micro emulsion, Eudragit S-100, 5-Fluorouracil

## Abstract

The objective of this study was to develop a novel bacterially-triggered micro-particular system of de-esterified tragacanth (DET) in combination with Eudragit S-100 coated capsules for colon drug delivery of 5-fluorouracil (5-FU) using microemulsion method. The loading study was conducted at different drug-to-polymer ratios and cross-linker concentrations. The maximum loading efficiency was achieved, 44.1% at 1:5 drug-to-polymer ratio and 0.7% cross-linker concentration. The FTIR results also confirmed the encapsulation of 5-FU in microspheres. The release profile was dependent on the cross-linker concentration, environmental pH, and presence of pectinase enzyme. Microspheres inserted into Eudragit S-100 coated capsules released less than 5% of the drug at stomach and small intestine pH levels, whereas 70% of the drug was released at colon pH levels, and about 25% of the drug did not release unless in the presence of pectinase enzyme. To omit burst release, microspheres were washed with water, and the release became pH independent, and was just achieved in the presence of pectinase enzyme. 5-FU loaded microspheres with an IC_50_ value of 80 µg/mL were as effective as the free drug on HT-29. Generally, the results demonstrated that drug-loaded microspheres inserted into Eudragit S-100 coated capsules can be effective for colon-targeted delivery.

## Introduction

Colorectal cancer (CRC) is the third most common malignant neoplasm worldwide (1) and surgery, radiation therapy, and chemotherapy are commonly used to treat it. The traditional dosage forms for treating CRC can deliver a drug to targeted sites; however, they also can reach other sites and cause adverse effects. Increasing the drug concentration at the targeted site is an important feature of site-specific delivery to the colon, which should reduce the required dose and incidence of side effects. The major obstacle for successfully targeting a drug to the colon via oral administration is avoiding absorption and degradation in the stomach and small intestine before arrival of the drug in the colon (2).

 Natural polysaccharides are strong candidates for colon drug delivery because they are undegraded in the stomach and small intestine, while they can be degraded by colon flora; the anaerobic bacteria in the colon can degrade polysaccharides with enzymes (3, 4). Among polysaccharides, ones with carboxylic acid groups are more attractive because their acidic groups can be deionized in pH of an acidic environment, which could reduce drug release in the stomach and increase the chance of efficient drug delivery into the colon, whereas pH is higher and polymer is more soluble (5, 6).

Tragacanth is a heterogeneous branched anionic polysaccharide from the pectin family that contains repeated galacturonic acid units in its backbone and galactose, fucose, and xylose in the side chains. De-esterified tragacanth (DET) is a water-soluble analog of tragacanth gum that contains a highly branched, high-molecular weight de-acetylated tragacanthic acid, which undergoes gelling with ionotropic complexation. Furthermore, it can be degraded by the pectinase of colon flora (7). 

Colon-specific delivery systems based on a single polysaccharide do not permit efficient targeted release. The composite of polysaccharides with other polymers and chemically modified forms of polysaccharides have eliminated the drawbacks of using a single polysaccharide (8). Different approaches, such as layer-by-layer coating and coating polysaccharides with pH sensitive polymers, have also been used to overcome these drawbacks (9-12). 

 Eudragit S-100 is a pH-sensitive anionic copolymer of methacrylic acid and methyl methacrylate. This polymer is soluble at pH 7 and above, and has been performed in various applications such as enteric coating materials and drug delivery systems (13-15).

 5-Fluorouracil (5-FU) is a pyrimidine analog and an antineoplastic drug that is used to treat metastatic cancers of the colon, breast, pancreas, head, neck, and ovary. The rapid absorption of 5-FU through blood capillaries into the systemic circulation together with subsequent enzymatic degradation in the liver results in a low drug concentration near the targeted site. The improvement in therapeutic efficacy and reducing the incidence of side effects can be achieved by controlled release of 5-FU (16). There are many reports on using polysaccharide micro- or nanoparticles for controlled release of 5-FU, or other drugs, in the colon (8, 17-20). To the best of our knowledge, this is the first report on using DET microspheres as colon-targeting drug carriers. Our objective in this research was to develop a novel bacterially triggered micro-particular system of tragacanth in combination with Eudragit S-100 coated capsules for colon drug delivery.

## Experimental

*Materials*

 Methanol, ethanol, sodium hydroxide, isooctane, and potassium di-hydrogen phosphate (KH_2_PO_4_) were purchased from Merck Chemical, Germany. Isopropyl alcohol was supplied from Applichem, Germany. Span 80 was purchased from Titrachem, Iran. Calcium chloride was purchased from Scharlau, European Union, Spain. Pectinase was supplied from Fluka, Switzerland. Eudragit S-100 was purchased from Evonik Industries, Parsippany, NJ, USA**. **Tragacanth gum was supplied from local market of Isfahan, Iran.

*Preparation of DET*

 DET has been prepared by de-esterification of tragacanth gum from *Astragalus-gossypinus* based of Fattahi *et al*. (7) with minor modification. Briefly, 2 g tragacanth was dispersed in 1 L sodium hydroxide (0.25 M) and stirred for 4 h at 4 °C, followed by precipitation of DET in 60% ethanol. Then precipitated DET was dissolved in deionized water. In order to remove sodium traces, acetic acid was added to the DET solution, and the solution was dialyzed using distilled water for 48 h. Finally, DET solution was freeze-dried for the next studies. 

*Molecular weight analysis of DET*

 Molecular weight of polymer was measured by DLS using Zetasizer (SZ, Malvern, UK). The average scattering intensity from five different concentrations of DET solutions (10-60 mg/mL) were recorded using the Malvern supplied ‘molecular weight’ operating procedure. Solvent of polymer, distilled water was used as the reference (21). 

*Preparation of microspheres*

**Figure 1:**
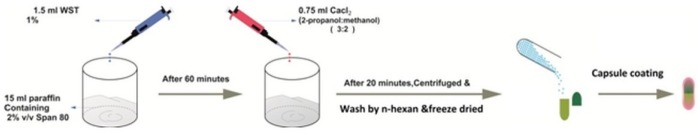
Schematic of DET microsphere preparation

One mL and half of DET 1% (with and without 5-FU) was emulsified in 15 mL liquid paraffin containing 2 vol% Span 80, using stirrer at 1300 rpm for 1 h. Then, 0.75 mL of CaCl_2_ (0.5-7%) in methanol/isopropyl alcohol (volume ratio of 2:3) was added dropwise to the emulsion, and the mixture was stirred for 20 min. Microparticles were collected by centrifuge and were washed by n-hexane for three times (Figure 1). In order to remove the burst release, sample of the best formulation was also rinsed with water thrice. Finally, microspheres were freeze-dried for further studies.

*Study of morphology and particle size*

The morphology of the cross-linked beads was examined using a scanning electron microscope (SEM, HIT-4160-02, Hitachi, Japan) and invert optical microscopy (AE31, Motic, China). 

For SEM study prior to examination, the samples were lyophilized, fixed on a brass stub, and coated with a gold-palladium layer under argon atmosphere using a gold sputter module in a high vacuum evaporator. For optical microscopy, the fresh samples were placed and analyzed directly in 6 wells plate. The average sizes of particles were calculated using ImageJ software (US National Institutes of Health, Maryland, USA) with n = 200. 

*FTIR study*

The microspheres were also characterized by Fourier Transform Infrared Spectroscopy (FTIR) (Irprestige-21, Shimadzo Co., Japan). For this purpose, FTIR spectra were acquired in transmission mode from DET powders, dried DET microspheres and 5-FU loaded microspheres. 

*Preparation of Eudragit S-100 coated capsules*

Hard gelatin capsules (size 1, Gelatin Capsule Iran, Iran) were manually filled with the freeze-dried microspheres. The capsules were then immersed in a methanol solution of Eudragit S-100 (15% w/v), followed by drying at room temperature using an air-blower. The procedure was repeated three times.

*Loading*

Specific amount of dry microspheres was vigorously stirred in a beaker containing 15 mL phosphate buffer solution at pH 8 to extract the drug from the microspheres. The solution was then filtered by 0.22 µm syringe filter and assayed by a UV spectrophotometer (UV 1240, Shimadzu, Japan) at 266 nm. The loading efficiency was calculated using Equation 1.

 (1) $$\mathrm{Percent of loading efficiency}(\mathrm{LE})=\frac{\mathrm{amount of drug in the microspheres}}{\mathrm{inetial amount of drug}}*100$$

For each formulation, determination was performed three times.

*Release of 5-FU*

The horizontal shaker method was used to study *in-vitro* release profile of water washed and unwashed microspheres and Eudragit S-100 coated capsules filed by microspheres. The temperature was kept at 37 °C and the stirring rate at 50 rpm. About 40 mg of 5-FU loaded microspheres were placed in the beaker containing simulated media, and samples were withdrawn at specified time intervals and centrifuged at 1000 rpm for 10 min; then the supernatant was filtered and spectrophotometrically assessed at 266 nm. The blank microspheres were taken as reference. Each experiment was repeated at least three times. For evaluation of stomach pH and colon pH effect on release rate of different formulations, release was investigated at pH 1.5 and pH 7.4, respectively. Then release of the optimum formulation was measured in the continuous model as follows to simulate GI pH; 1 h at pH 1.5 (gastric media), 2 h at pH 4.5 (initial part of small intestine), 2 h at pH 6.5 (end part of small intestine) and finally 4 h at pH 7.4 (the colon area). In order to verify the release profile in presence of bacterial enzyme, 10 mM phosphate buffer (pH 7.4) containing 2% pectinase enzyme (Fluka, Switzerland) was used as dissolution medium and the assay was performed as described above. 

*Cell culture*

HT-29 cell line (a human colorectal adenocarcinoma cell line with epithelial morphology) was obtained from Pasteur Institute (Tehran, Iran). These cells are sensitive to 5-FU which is one of the standard treatment options for colorectal cancer. HT-29 cells were grown in RPMI 1640 medium (Gibco, Scotland) supplemented with 10% Fetal Bovine Serum (FBS, Gibco, Scotland) and penicillin/streptomycin (50 IU/mL, 50 µg/mL) at 37 °C in a humidified atmosphere of 5% CO_2_. The cells were sub-cultured regularly using trypsin/EDTA. 

*Cell viability assay*

The thiazolyl blue (MTT) assay has been used in many experiments for assessment of cell viability, and this reaction is used as the end point in a rapid drug-screening assay. Briefly, the cells were seeded at density of 1 × 10^5^ cells/mL in 96-well tissue culture plates and were re-suspended in 10 mL complete culture medium and allowed to attach for 24 h. After this period, the cells were incubated with increased concentrations of 5-FU (0.01, 0.01, 0.1, 1, 50, and 100 µg/mL) for 48 h, separately. MTT solution (20 µL) was then added to each well and plate was incubated for 3 h at 37 °C. During this period, living cells produced blue insoluble formazan from the yellow soluble MTT. The reaction was stopped by removing medium, washing wells by phosphate buffer solution and adding DMSO (150 µL/wells). The contents of the wells were dissolved during 2–3 min. Absorbance was determined on an ELISA plate reader (Biotek, H1M, USA) with a test wavelength of 570 nm and a reference wavelength of 630 nm to obtain sample signal (OD570-OD630).

*Data analysis*

Statistical evaluation of data was performed using an analysis of variance (ANOVA); in all cases, a value of *p* ≤ 0.05 was accepted as significant.

## Results and Discussion

*Properties of DET*

Fattahi *et al.* reported that DET could not produce a stable gel with calcium cations (7). In that study, HCl was used to neutralize excess NaOH, and the solution was precipitated by ethanol. In this procedure, a high amount of sodium chloride (NaCl) was produced, which is a low soluble salt in ethanol. The presence of NaCl in the final product could affect the interactions of DET and calcium and destabilize the gel. To solve this problem, DET solution was dialyzed to remove small molecular weight impurities and salts, and the resulting DET would form a stable gel with calcium ions. The average molecular weight of DET measured by Zetasizer was 595 ± 29.7 kDa.

*Particle size and morphology*

**Figure 2:**
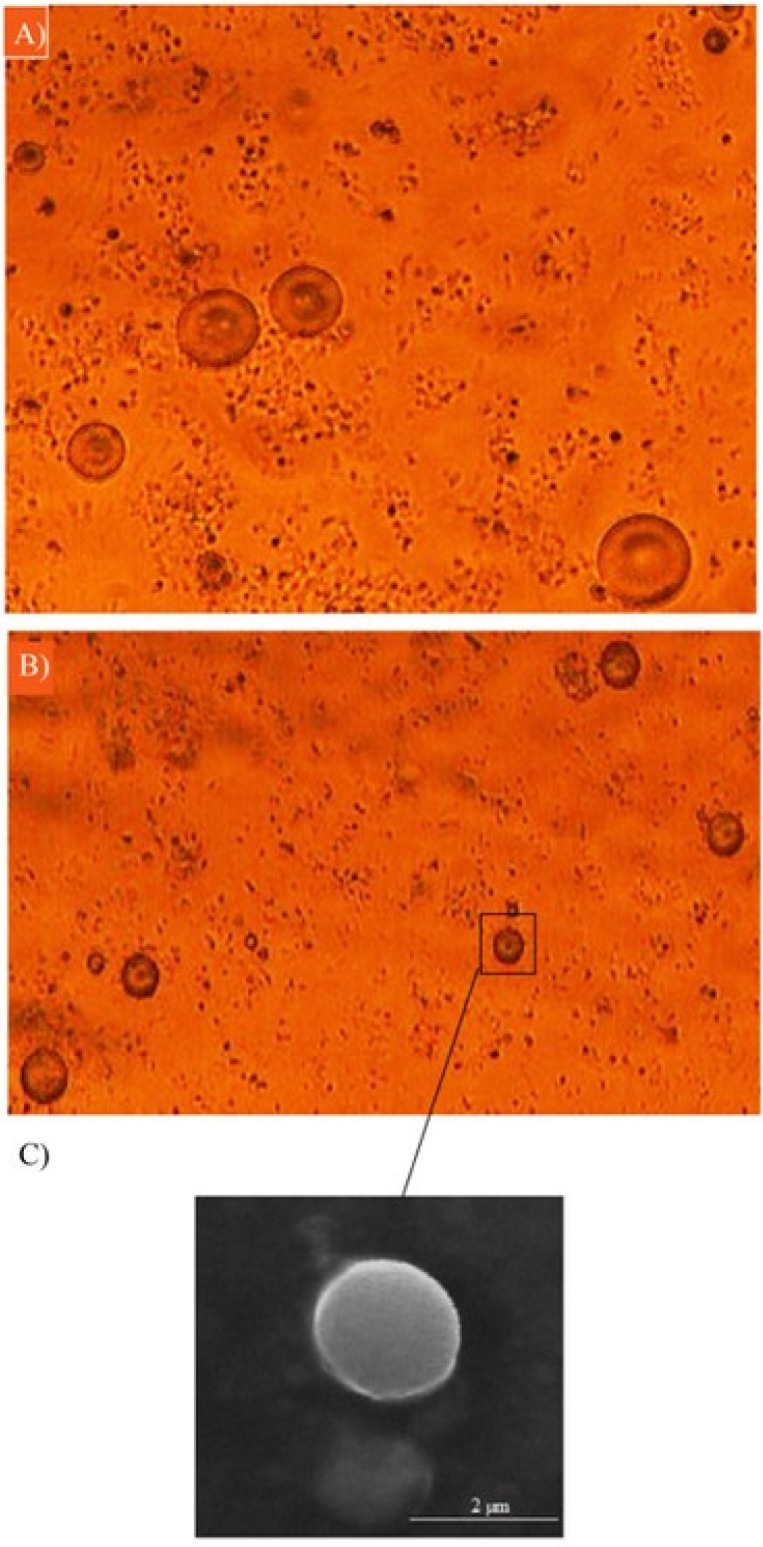
Optical microscopy images of DET microspheres cross-linked by (A) 0.5% and (B) 5% CaCl_2_, (C) SEM image of DET microsphere cross-linked by 5% CaCl_2_

**Figure 3:**
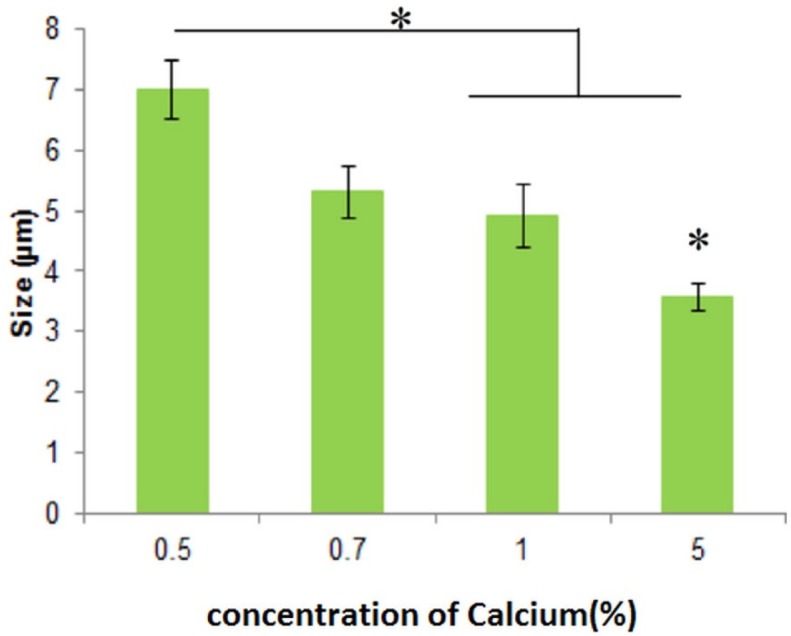
Effect of cross-linker (CaCl_2_) concentration on particle size of DET microspheres; Significance was calculated by ANOVA (*p ≤ 0.05

As can be seen in Figure 2, the microemulsion method synthesized spherical DET particles. Optical microscopy images (Figures 2A-B) revealed that the amount of the cross-linker did not affect morphology, whereas increasing the cross-linker concentration from 0.5% to 5% significantly reduced the particle size. Although increasing the concentration of cross-linker causes the size of microspheres to increase, it could also decrease the loading efficiency, and could reduce the rate of drug release. The viscosity of solution also affects the size of microspheres. Increasing the viscosity of microspheres leads to increasing the size of particles. Increasing the viscosity of solution could be because of a higher concentration of DET (22, 23). The particles were spherical with sizes ranging from 3.55 ± 0.22 to 7.0 ± 0.478 µm (Figure 3) for different formulations. The spherical structure of DET particles could be observed under the scanning electron microscope (SEM), where the smooth surface was more evident (Figure 2C).

*FTIR analysis*

**Figure 4:**
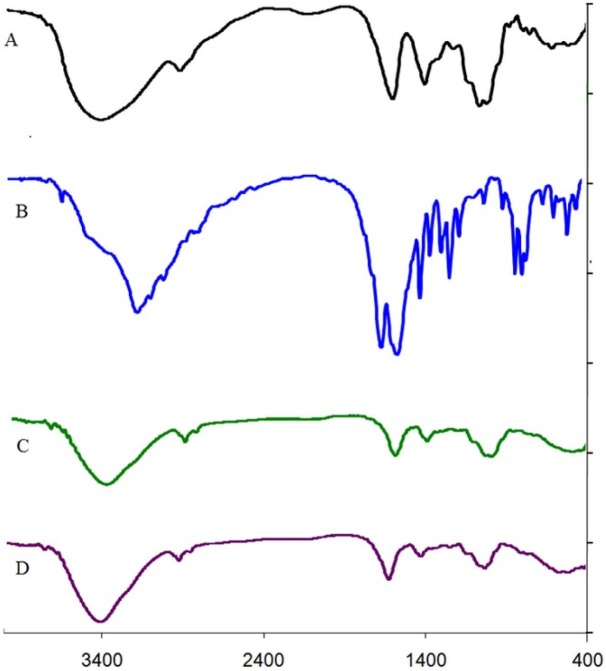
FTIR spectra of (A) DET, (B) 5-FU, (C) microspheres and (D) 5-FU loaded microspheres

The characteristic peaks of DET, 5-FU, DET microspheres, and 5-FU loaded microspheres are shown in Figure 4. The DET peaks revealed asymmetric stretching of COO (attributed to sodium salt of carboxylic acid) at 1,616.35 cm^−1^ and symmetric stretching of COO at 1,415.75 cm^−1^ (Figure 4A). The peak attributed to O-H stretching in DET at 3,500 cm^−1^ was narrow, and its intensity increased after cross-linking with calcium ions because of decrease in hydrogen bond (Figures 4A and C). In the spectrum of 5-FU, peaks at 1,654 cm^−1^ and 1,554 cm^−1 ^associated overlap stretching vibration absorption of carbonyl and C=C, and the peaks at 550 and 650 cm^−1^are attributed to the C-F functional group and OOP (out-of-plane) peak, respectively (Figure 4B). The spectrum of 5-FU loaded microspheres showed that characteristic peaks of 5-FU. The peak at 1,627.29 cm^−1^ was attributed to the secondary amine, and carboxyl group of 5-FU together with the carboxyl group of tragacanth. The bands at 1,342.46–1,369.46 cm^−1^ were related to vibrations of the pyrimidine compound, and the peak observed at 1,253.57 cm^−1^ was attributed to C-F stretching, that provided evidence of 5-FU encapsulation in microspheres (Figure 4D). In this spectrum (4D), intensity of peak at 1,627 cm^−1^ was higher than that of peak at 1,050 cm^−1^ in comparison with the DET spectrum; this could be related to the absorption of the carbonyl group of loaded 5-FU (Figures 4C and 4D).

*Loading efficacy (LE)*

LE is a critical parameter in drug delivery by micro- and nano-hydrogels. The presence of a high amount of water inside and outside of microspheres makes it difficult to encapsulate a high percent of the drug in the microspheres (9). One way to improve LE is to use a minimum amount of water in the preparation procedure. Other parameters that control LE are the drug-to-polymer ratio and percentage of cross-linkers. To evaluate the effect of the drug-to-polymer ratio, LE at ratios of 1:1 to 1:5 in the constant concentrations of DET (1%) and CaCl_2_ (5%), was studied. Although LE increased by reducing the drug-to-polymer ratio from 1:1 to 1:5, there was no significant difference between the ratios 1:2 and 1:5 (Table 1). For further studies and evaluating the effect of cross-linker concentration on LE, the formulation with a drug-to-polymer ratio of 1:5 was chosen. As shown in Table 1, the loading efficiency decreased by increasing the concentration of the cross-linker, but no statistically significant difference was observed after increasing the cross-linker concentration from 0.5% to 5%, which shows that the effect of cross-linker concentration on LE is negligible.

*In-vitro release study*

**Figure 5:**
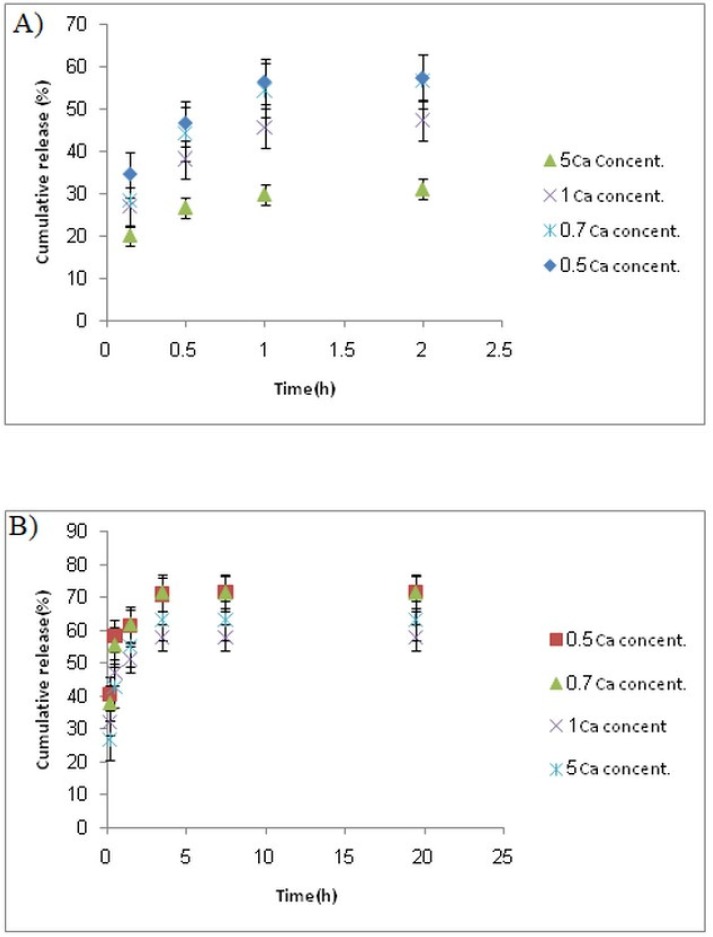
Cumulative release of 5-FU from microspheres prepared by different percent of CaCl_2_ (0.5-7%) at (A) pH 1.5 and (B) pH 7.4

Colon-targeted drug delivery systems must protect the drug during its transport through the stomach and small intestine, but allow fast release on entry into the colon. Release profiles of samples at stomach and colon pH levels (Figures 5A-B) indicated that release of 5-FU at pH 1.5 (stomach pH) was slower than that at pH 7.4 (colon pH) for all formulations.

**Figure 6.**
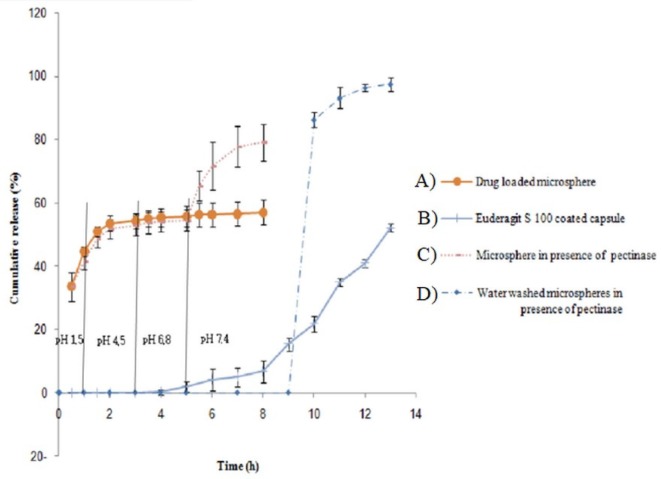
*In-vitro* 5-FU release of DET microsphere in progressive pH medium: (A) DET microspheres, (B) Eudragit S-100 coated capsule containing DET microspheres, (C) DET microsphere in presence of pectinase, (D) water washed DET microspheres in presence of pectinase

This was attributed to a lower swell ability of DET hydrogel at an acidic pH because of carboxylic acid deionization. At both pH values, the release rate decreased with increasing the CaCl_2_ concentration. The release profile revealed a burst release at both pH levels, whereas 30%–55% of 5-FU was released at stomach pH, and 50%–70% was released at colon pH. The remaining amount was not released even after 20 h. This burst and fast release might be attributed to a portion of drug that was loaded on the surface of the microspheres. We proposed two strategies to overcome this drawback; the first method was filling the hard gelatin capsule with microspheres and coated capsules with Eudragit S-100, which is a pH sensitive polymer and is soluble at pH 7 or higher. Using this approach, less than 5% of the 5-FU was released when the capsule incubated in the stomach and small intestine pH levels; whereas approximately 70% of the drug was released within 10 h in a colon pH level, around 25% of the drug was not released, even at a pH 7.4 (Figure 6). This could be related to Van der Waals’ interactions between 5-FU and DET.

In order to simulate the enzymatic actions in the colon, pectinase was added to the medium. Release of 5-FU in the presence of pectinase was significantly increased. Considering encapsulating DET microspheres in an enteric-coated capsules and pectinase effect, 5-FU release can be controlled and biphasic release can be achieved. Quick release can be achieved on capsule entry into the colon, which is related to the burst release of the drug from the microsphere surfaces and the release after pectinase-mediated DET degradation. Another strategy for this problem is washing microspheres by water to remove burst release. Although loading efficacy decreased, drug release was pH independent by this method. The release of the drug could be achieved just in the presence of pectinase enzyme and no drug was released at different pH unless in the presence of pectinase enzyme. These enzyme-sensitive microspheres can be used as a suitable option for a colon-targeted delivery system. 

**Table 1:** Effect of cross-linker concentration, drug to polymer ratio and washing by water on drug loading and loading efficiency.

Cross linker (%)	5	5	5	0.5	0.7	1	5
Drug: polymer ratio	1: 1	1:2	1:5	1:5	1:5	1:5	1:5 (washed by water)
Loading Efficiency	35.4 ± 0.58	40 ± 13.18	42.3 ± 11	43.4 ± 5.5	44.1 ± 7.95	42.5 ± 13.24	20.71 ± 0.82

**Figure 7:**
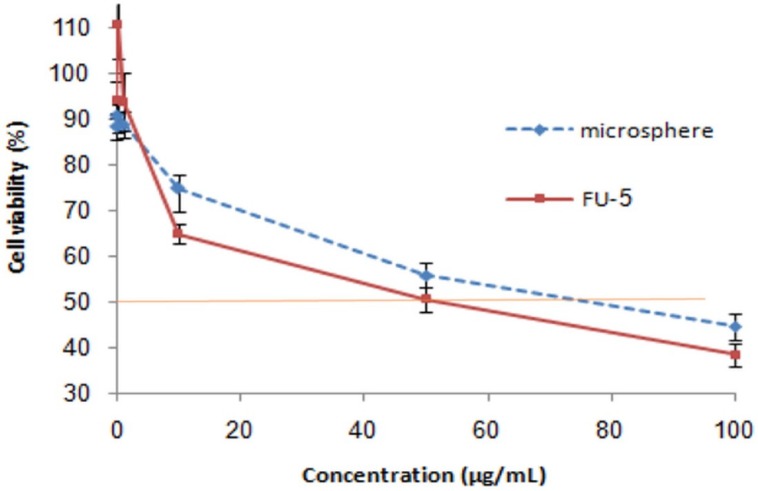
Relative cell viability of free 5-FU and drug loaded microspheres (produced by 1% DET, 5% CaCl_2_ and ratio of drug to polymer 1:5) on HT29 cell line. The relative cell viability read for the control (tissue culture polystyrene from culture plates) after 48 h of incubation was taken as the reference (100%). Significance was calculated by ANOVA (*p ≤ 0.05

*Cytotoxicity*

The viability of HT29 cells in the presence of 5-FU loaded microspheres (produced by 1% DET, 5% CaCl_2_, and containing 1:5 ratio of drug to polymer) and free drug, was investigated to evaluate the potency of the loaded microspheres. As seen in Figure 7, neither the free drug nor the loaded drug showed efficient cytotoxicity at 0.01–1 µg/mL. A considerable reduction in cell viability was observed when HT29 cells were incubated with either 100 µg/mL of 5-FU or with microspheres containing 100 µg/mL of 5-FU. The IC_50_ of the free drug and drug-loaded microspheres was 50 and 80 µg/mL, respectively. A higher IC_50_ for microspheres could be attributed to the profile of release, whereas around 25% of the drug could not be released at pH 7.4. Considering the low ability of cells to uptake microspheres and lack of pectinase in cell culture medium, the unreleased drug could not be available for cells, which reduced the potency of microspheres in comparison with the free drug.

## Conclusion

In the present study and for the first time, microspheres of DET were prepared and 5-FU, a hydrophilic anticancer drug model, was successfully loaded into the microspheres. The spherical particles with sizes ranging from 3.55 ± 0.22 to 7.0 ± 0.478 µm were obtained in this study. 

By increasing the cross-linker concentration, it was found that particle size could be smaller at higher concentrations, but no statistically significant difference in LE was observed after increasing the cross- linker concentration. The LE was affected by the drug-to-polymer ratio, which increased by reducing this ratio. It was found that up to 44% LE could be attained using microspheres with 1:5 drug-to-polymer ratios and 0.7% cross-linker. 

The presence of the drug in microspheres was also examined using FTIR analysis and the FTIR spectra revealed the encapsulation of 5-FU in DET microspheres. The release of 5-FU at stomach pH was observed to be slower than at colon pH, but the burst release at both pH levels was observed. In order to omit or decrease the burst release, the hard gelatin capsule was filled with microspheres, and coated with a pH-sensitive polymer (Eudragit S-100). Using this method, less than 5% drug was released at stomach and intestine pH, whereas 70% release was obtained at colon pH. Furthermore, washing the microspheres with water was another way to overcome the burst release. This method resulted in a pH-independent and enzyme-sensitive drug release profile. Altogether, DET microspheres together with Eudragit S-100 coated capsules can be an effective system for targeting drug delivery to the colon.
